# Combined surgical and radiosurgical treatment for a symptomatic cervical metastasis in a case of malignant paraganglioma: a case report

**DOI:** 10.1186/1756-0500-6-494

**Published:** 2013-12-01

**Authors:** Ken Sasaki, Hiroyuki Inose, Shigenori Kawabata, Toshitaka Yoshii, Tsuyoshi Kato, Masanori Saito, Atsushi Okawa

**Affiliations:** 1 Department of Orthopedics, Tokyo Medical and Dental University, 1-5-45, Yushima, Bunkyo-ku, Tokyo 108-0075, Japan

**Keywords:** Malignant paraganglioma, Metastasis, Radiosurgery, Surgery

## Abstract

**Background:**

Paragangliomas of the head and neck are rare tumors. Moreover, malignant paragangliomas of the cervical spine are extremely rare. Currently, the combination of curative surgical resection and secondary adjuvant radiotherapy is the gold standard for treating symptomatic malignant paragangliomas. However, traditional treatments for malignant paraganglioma remain unsuccessful. The purpose of this study is to report an exceedingly rare case involving cervical metastasis of a malignant paraganglioma.

**Case presentation:**

In this case report, we present a case involving a 72-year-old male with a history of paraganglioma of the neck. He had been experiencing bilateral shoulder pain, neck pain and weakness in the upper extremities for more than six months. Magnetic resonance imaging of the cervical spine revealed a metastasis at C4 with severe vertebral body destruction. To avoid serious complications associated with surgical resection, CyberKnife® radiosurgery (Accuray, Inc., Sunnyvale, CA, USA) was performed on the parapharyngeal and cervical lesions. A secondary surgery, which involved a posterior laminectomy at C3-6 and posterior fusion at C1-T1, was performed two weeks after the radiosurgery. A histological examination of the surgical specimen demonstrated a malignant paraganglioma. The patient regained strength in all extremities in the postoperative field, and his pain was dramatically reduced. A magnetic resonance imaging study performed three months after the surgery showed a reduced tumor size and spinal cord decompression.

**Conclusion:**

This case study is the first report of a patient with symptomatic cervical metastasis of a malignant paraganglioma treated with a combination of radiosurgery and posterior spinal surgery. Although the optimal treatment for these conditions remains unclear, concomitant treatment with radiosurgery and reconstructive surgery appeared to be both safe and effective in this challenging case.

## Background

Paragangliomas are highly vascular tumors that arise from paraganglia. Of these, head and neck paragangliomas are rare, accounting for about 0.03% of all tumors [1]. Malignant paragangliomas are exceedingly rare. Currently, the combination of curative surgical resection and secondary adjuvant radiation is the gold standard for treating symptomatic malignant paragangliomas [2,3]. However, the postoperative complications are considerable. To our knowledge, the combined surgical and radiosurgical treatment for malignant paragangliomas described in this study have not been reported previously. This report describes a patient with severe neck pain and myelopathy.

## Case presentation

**Figure 1:**
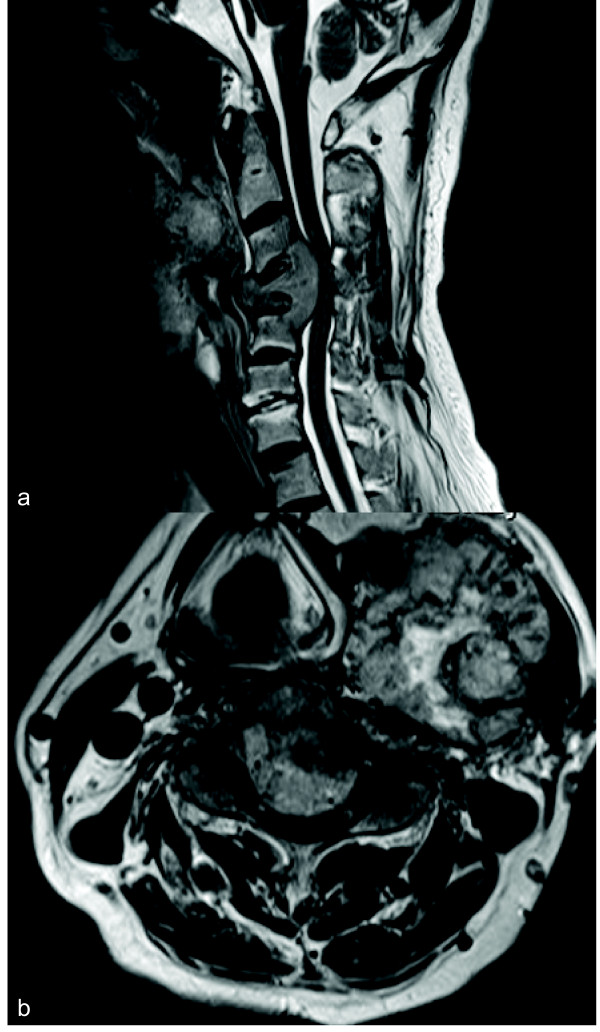
Sagittal (a) and axial (b) T2-weighted magnetic resonance image displaying marked C4 destruction and severe canal stenosis.

**Figure 2:**
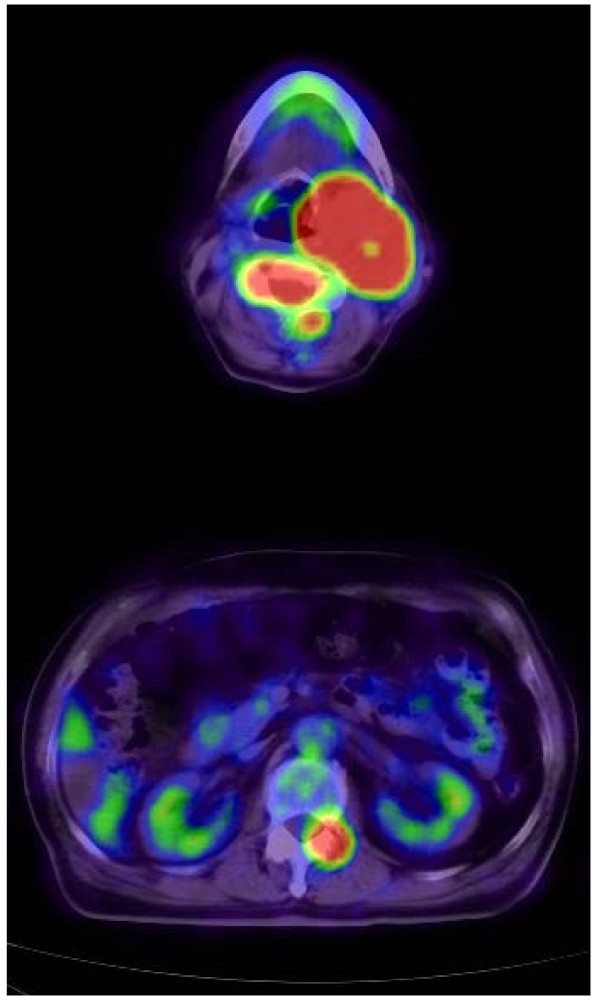
Positron emission tomography-computed tomography showing pathological fluorodeoxyglucose uptake in the neck (top) and in the C4 (top), L1 (bottom) vertebral bodies.

We report a rare case of symptomatic cervical metastasis of a malignant paraganglioma in a 72-year-old male. Five years prior to presentation, the patient visited another hospital presenting with neck swelling and hoarseness. He was diagnosed with paraganglioma of the neck, but an operation was not planned at that time. Six months prior to presentation, the patient’s chief complaints were bilateral shoulder pain, severe neck pain and weakness of the upper extremities. Because of the continual pain, he was referred to us for a second opinion. A physical examination revealed clumsiness in both hands and muscle weakness in the upper extremities. Cervical roentgenograms revealed a lytic lesion of the C3,4,5 vertebral body. Cervical computerized tomography (CT) revealed destruction of the C3,4,5 vertebral body and C4 lamina. Magnetic resonance imaging (MRI) (Figure 1) demonstrated severe canal stenosis at the C4 level, with spinal cord deformation. An MRI of the neck upon admission showed a large soft-tissue tumor in the parapharyngeal space, which was adherent to the left carotid artery. A positron emission tomography (PET) scan (Figure 2) revealed lytic lesions of the C4 and L1 vertebrae and a parapharyngeal space lesion near the carotid body.

**Figure 3:**
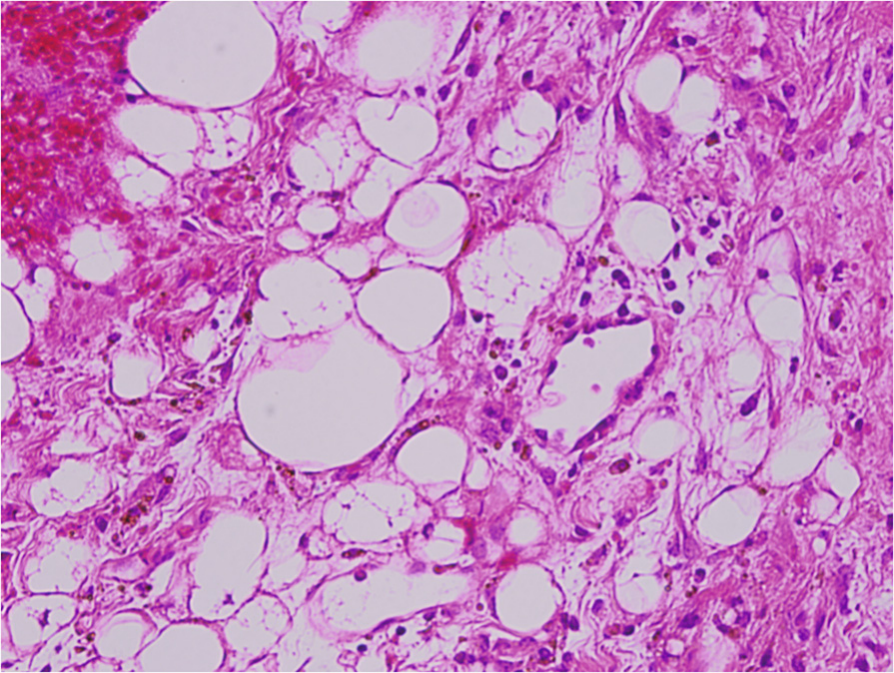
Microscopic findings revealing polygonal cells with eosinophilic cytoplasms within a prominent vascular component (the typical zellballen structures, H&E 200×).

**Figure 4:**
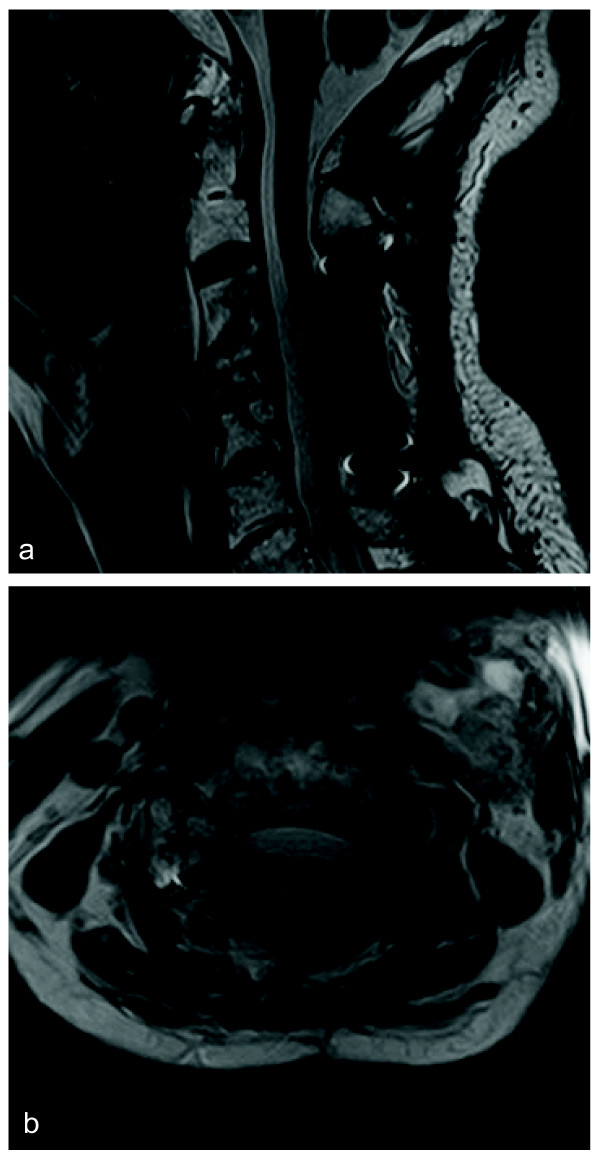
Postoperative sagittal (a) and axial (b) T2-weighted magnetic resonance image showing a reduced tumor size and spinal cord decompression.

As the tumor was extremely large and close to the artery and nervous tissue, the head and neck surgeons avoided this treatment route. The conventional anterior approach to the vertebral body was also rendered impossible because of the obstruction of the primary neck tumor. Therefore, CyberKnife® radiosurgery (Accuray, Inc.) was employed as the primary treatment for the cervical and parapharyngeal lesions. Even after the radiosurgery, the pain and weakness in the upper limbs persisted. We concluded that it would take some time for the effect of radiosurgery to appear, as his severe neck pain was caused by the destruction of vertebrae. Consequently, a C3-6 laminectomy and C1-T1 fusion using pedicle screw fixation was also performed. The highly vascularized tumor on the C4 lamina was removed and sent to the pathology department. A histological examination (Figure 3) revealed a malignant paraganglioma. Immediately after the second operation, the patient displayed significant improvement in pain and upper-extremity weakness without any complications. As for the metastasis in the lumbar lesion, the patient had yet to show symptoms, a regular course of radiation was administered. Three months after the second operation, an MRI (Figure 4) confirmed that the tumor had shrunk, and the spinal cord compression had disappeared. The last follow-up (six months after the second operation) demonstrated complete recovery of the upper-extremity strength.

## Discussion

Paraganglioma is a rare and slow-growing tumor that arises from extra-adrenal chromaffin cells and accounts for 0.3% of all neoplasms [4]. Malignant paraganglioma accounts for 3% of paragangliomas. Malignant paragangliomas are defined by findings of local recurrence or presence of metastatic spread of tumors. Vertebral metastases of malignant paraganglioma are exceedingly uncommon. Amongst these, cervical vertebral metastases are the rarest [3]. To the best of our knowledge, only ten isolated reports exist [2,3,5-7]. The symptoms caused by cervical spinal metastases are predominantly the consequence of the tumor’s mass effect on local structures, such as the spinal cord and vertebral body.

Paraganglioma can be diagnosed by measuring the catecholamines and metanephrines in the plasma or urine. CT or MRI can identify the location of the paraganglioma. Additionally, ^131^I-metaiodobenzylguanidine (MIBG), a precursor of noradrenaline, scintigraphy has a sensitivity of 77%–90% and a specificity of 95%–100% in detecting paragangliomas [8]. However, while reduced ^131^I-MIBG sensitivity has been demonstrated in some cases, PET has been found to be superior to ^131^I-MIBG scintigraphy [9,10]. Our case showed a pathological ^18^ F-fluorodeoxyglucose (FDG) uptake in the neck and in the C4, L1 vertebral bodies on PET.

Pathologically, a *Ki-67 index* <1% is considered a useful parameter for predicting benign potential [11]. In our case, the histological findings were compatible with malignant paraganglioma, showing a *Ki-67 index* > 1%, polygonal cells with eosinophilic cytoplasm in a prominent vascular component (the typical Zellballen structures), and lastly positive staining for chromogranin, synaptophysin and CD56.

Currently, the combination of curative surgical resection and secondary adjuvant radiotherapy is the gold standard for treating symptomatic malignant paragangliomas [2,3]. Surgical resection is effective; however, these procedures contain potential risks, such as massive bleeding, spinal cord damage and peripheral nerve damage. To date, I^131^-MIBG remains the only other non-surgical treatment that has produced tumor responses >30% [10]. However, this treatment has not been confirmed to be effective in tumors that lack MIBG uptake. Most importantly, the procedure is not curative.

Recently, radiosurgery has emerged as an effective tool for managing paraganglioma [12]. Radiosurgery delivers external radiation in a focused manner, allowing for much higer doses to be given with fewer side effects to the surrounding normal tissue [13]. Guss *et al.* have reported that 97% of patients with paraganglioma achieve tumor control through radiosurgery, the results are comparable to that of surgery [14,15]. However, no studies have specifically investigated the use of CyberKnife® radiosurgery for a malignant paraganglioma [16]. Thus, its application has not been well established. In our case, according to the MRIs before and after radiosurgery, tumor mass was markedly reduced. If the radiosurgery had such a great effect on tumor control, there should be the question that posterior fusion surgery might not have been needed. However, even after the radiosurgery, his severe neck pain and muscle weakness continued. We thought that these symptoms were caused by the instability of the spine due to the destruction of the vertebral body and continuous spinal cord compression. Therefore, we think this case also needed the posterior decompression and fusion surgery.

## Conclusions

In summary, we report a rare case of symptomatic cervical metastasis of a malignant paraganglioma. Radiosurgery followed by dorsal spinal surgery led to rapid improvement of the paresis and pain. Postoperative imaging findings and clinical outcome revealed the safety and effectiveness of combined surgery and radiosurgery. However, additional follow-ups are necessary to detect local recurrence and the spread of other lesions.

## Consent

Written informed consent was obtained from the patient for publication in this case report and any accompanying images. A copy of the written consent is available for review by the Editor-in-Chief of this journal.

## Abbreviations

CT: Computed tomography; MRI: Magnetic resonance imaging; FDG: Fluorodeoxyglucose; PET: Positron emission tomography; MIBG: Metaiodobenzylguanidine.

## Competing interests

The authors report no conflict of interest concerning the materials or methods used in this study or the findings specified in this paper.

## Authors’ contributions

KS analyzed the treatment and contributed to the final draft of the manuscript. HI planned the treatment, performed the operative procedure, and drafted the manuscript. MS carried out final preparation of the manuscript. SK, TY, TK, and AO planned the treatment and contributed to the final draft of the manuscript. All authors read and approved the final manuscript.
